# Incidence of Pharyngocutaneous Fistula After Total Laryngectomy and Its Relationship With the Shapes of Mucosa Closure: A Meta-Analysis

**DOI:** 10.7759/cureus.28822

**Published:** 2022-09-06

**Authors:** Adit Chotipanich, Sombat Wongmanee

**Affiliations:** 1 Department of Otolaryngology-Head and Neck Surgery, Chonburi Cancer Hospital, Chonburi, THA

**Keywords:** total laryngectomy, horizontal closure, vertical closure, t-shaped closure, pharyngocutaneous fistula

## Abstract

Background and objective

Pharyngocutaneous fistula is the most common complication after total laryngectomy. The aim of this study was to examine the incidence of fistula and the association between fistula and the shape of mucosal closure (T-shaped, vertical, or horizontal closure).

Method

A search of English language databases from 1979 to 2021 was undertaken for studies of total laryngectomy that commented on pharyngeal closure techniques and fistulas. Pooled estimates for fistula incidence and odds ratios were calculated.

Results

A total of 24 retrospective studies were included. The pooled fistula rates in primary total laryngectomy were 19.9% with T-shaped closure, 16.1% with vertical closure, and 16.4% with horizontal closure. In salvage total laryngectomy, the pooled fistula rates were 35.1%, 36.1%, and 17.9% with T-shaped, vertical, and horizontal closure, respectively. In the analysis of association, the risk of fistula formation in the T-shaped closure was not significantly different compared to that in the vertical closure, (odds ratio, 0.96; 95% confidence interval, 0.46-2.00). The horizontal closure, when compared to vertical closure, was significantly associated with lower risk of fistula formation (odds ratio, 0.31; 95% confidence interval, 0.12-0.78), but had nonsignificant lower risk of fistula formation when compared to the T-shaped closure (odds ratio, 0.46; 95% confidence interval, 0.19-1.12).

Conclusion

Horizontal closure seems to be the best closure shape for primary repair after total laryngectomy. However, analysis bias may have occurred because of the lack of well-controlled studies.

## Introduction and background

Total laryngectomy is a commonly used curative procedure for the treatment of advanced and recurrent tumors, and other noncancerous conditions of the larynx. After complete removal of the larynx, the resulting defect of the pharynx is repaired, creating the so-called neopharynx. Ideally, the pharyngeal reconstruction must be watertight to avoid leakage, sufficiently large to allow food passage, and capable of accommodating voice rehabilitation. Complications related to neopharyngeal reconstruction include salivary fistula, diverticulum, and stenosis.

Pharyngocutaneous fistula is the most common complication related to neopharyngeal reconstruction [1]. Previous radiotherapy, T-stage, flap reconstruction, and underlying health conditions such as smoking, anemia, and malnutrition, are known risk factors for post-operative fistula [1]. Surgical techniques during neopharyngeal reconstruction may affect the occurrence of the fistula, but this has not been thoroughly researched [2]. 

When possible, primary closure is the first choice for neopharyngeal reconstruction because it has better function and is less complicated [3]. The mucosal repair in primary closure can be either straight (vertical or horizontal) or a T (or Y)-shaped line. The best pharyngeal reconstruction technique and the shapes of pharyngeal closure to be used remain disputed [2].

this study aimed to pool the available data on the fistula incidence of each closure shape and to determine the association between these closures and risk of fistula formation.

## Review

Methods

Search Strategy

A search of English language databases from 1979 to 2021 was undertaken. The studies were retrieved from The Cochrane Library, PubMed, and Google Scholar databases using the free text terms, “total laryngectomy,” “fistula,” and “closure.” Hand searches of the reference lists of the included articles were also performed.

Selection Criteria

Two authors independently assessed the titles and abstracts for eligibility, and full-text articles were reviewed for closer examination. Studies were included if the following criteria were fulfilled: (1) the study reported on fistula incidence after total laryngectomy with primary closure reconstruction, (2) types of closure and number of cases were mentioned in detail. (3) For pooled analysis of incidence, the fistula outcomes between primary or salvage surgery were clearly specified. (4) For association analysis, the study contained two or more comparative groups with different closure shapes. Exclusion criteria were as follows: (1) usage of primary closure with flap reinforcement, (2) usage of staple closure or salivary bypass tube, and (3) case reports and case series.

Quality Assessment

For pooled analysis of incidence, studies were graded using six criteria based on the STROBE statement [4]. The criteria included (1) clear description of the study setting, (2) clear description of the study population, (3) details on fistula diagnosis, (4) whether informed consent was provided by participants, (5) whether the study examined consecutive patients, and (6) whether the results of the study can be generalized to the wider community. A study was considered to be of poor quality if it did not meet more than two criteria. The Newcastle-Ottawa Scale was used to assess the quality of cohort studies [5]. A study with a score below 4 was considered to be high risk for bias.

Statistical Analysis

Meta-analysis was performed using Review Manager software (version 5.2). The random effects and fixed effects models were used, but the random effects model was preferred because of the diversity of surgical techniques and patients among the included studies. Studies with low quality or high risk of bias were not included in the pooled analysis. The pooled incidence rates of fistulas after primary and salvage total laryngectomy were calculated separately. Differences of fistula outcome between closure shapes were considered significant if a 95% confidence interval did not include the null value.

Results

**Figure 1:**
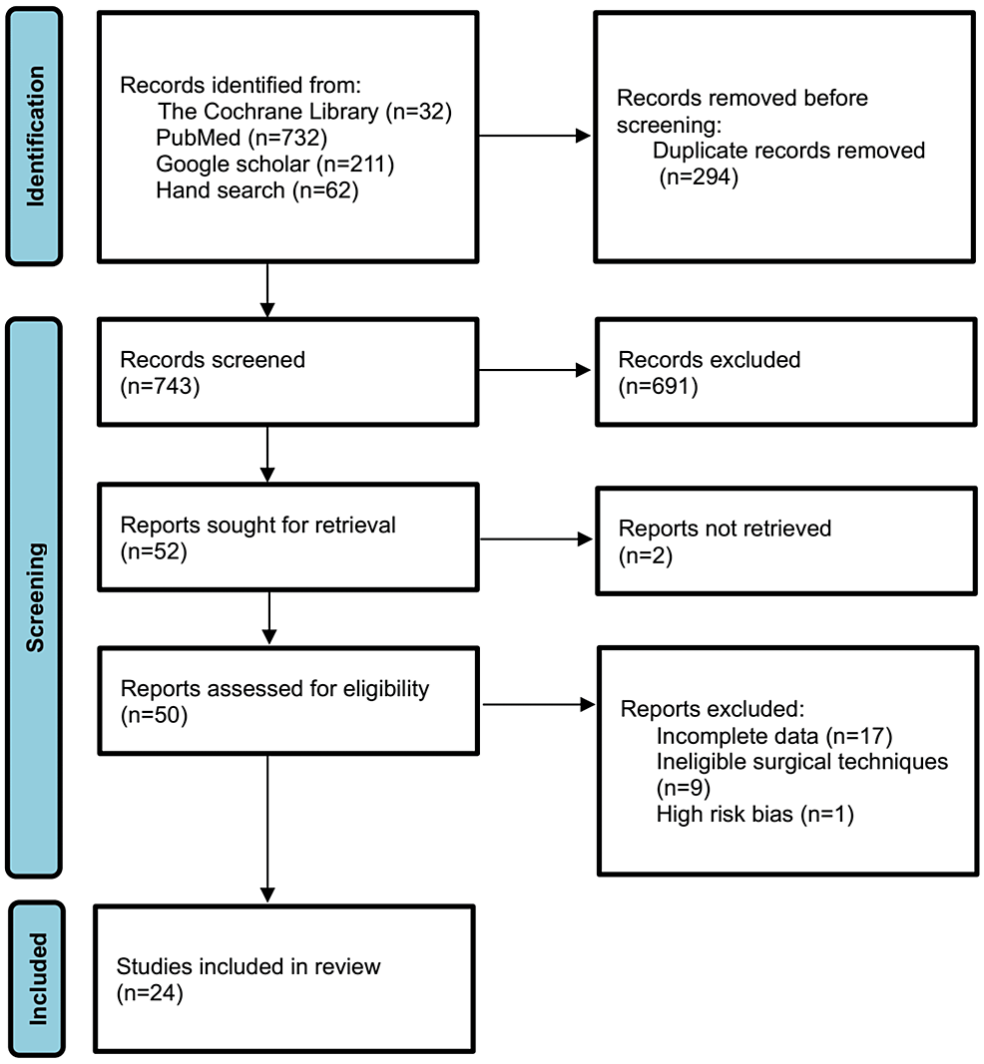
PRISMA 2020 flow diagram PRISMA: Preferred Reporting Items for Systematic reviews and Meta-Analyses

Figure 1 depicts the Preferred Reporting Items for Systematic reviews and Meta-Analyses (PRISMA) flow diagram. A total of 24 studies met the inclusion criteria. Of these, 17 were eligible for pooled analysis of fistula incidence and 14 for the analysis of association. All studies were retrospective [6-29].

Tables 1, 2 summarize the pooled analysis of fistula incidence after primary total laryngectomy and salvage total laryngectomy, respectively. Vertical closure had the lowest pooled fistula rate in primary surgery, while horizontal closure had the lowest pooled fistula rate in salvage surgery. 

**Table 1:** Summary of studies included for analysis of pooled estimates for fistular rates in primary total laryngectomy. RF: random-effects model; FE: fixed-effects model; CI: confidence interval

Types of closure	Case	Fistula (%)	Surgical technique (suture of mucosa layer, number of layers, and suture material)	Quality assessment
T-shaped closure				
Davis et al. (1982) [7]	15	13.3%	Interrupted or Connell suture, three-layer closure with catgut	5/6
Virtaniemi et al. (2001) [10]	95	9.5%	Connell suture, two-layer closure with Dexon	4/6
Markou et al. (2004) [11]	308	13.3%	Interrupted suture, two-layer closure	4/6
White et al. (2012) [13]	62	24.2%	Connell or Lembert suture, two-layer closure	4/6
Deniz et al. (2015) [15]	7	57.1%	Interrupted suture with polyglactin 910	4/6
Busoni et al. (2015) [16]	163	19.0%	Single-layer closure	4/6
Süslü et al. (2016) [19]	22	13.6%	Continuous suture, three-layer closure with polyglactin 910	5/6
Nitassi et al. (2016) [20]	51	35.3%	Not available	4/6
Walton et al. (2018) [24]	39	2.6%	Not available	4/6
Govindasamy G (2018) [25]	5	20.0%	Not available	4/6
Kitano et al. (2021) [29]	28	10.7%	Continuous suture, two-layer closure with Polydioxanone	4/6
Vertical closure				
Davis et al. (1982) [7]	12	25.0%	Interrupted or Connell suture, three-layer closure with catgut	5/6
Mohamed et al. (2014) [14]	66	4.5%	Connell suture, two-layer closure with polyglactin 910	4/6
Deniz et al. (2015) [15]	13	0	Cushing suture with polyglactin 910	4/6
Nitassi et al. (2016) [20]	21	38.1%	Not available	4/6
Walton et al. (2018) [24]	81	18.5%	Not available	4/6
Ogunkeyede et al. (2020) [27]	39	10.3%	Continuous suture, two-layer closure with polyglactin 910	4/6
Horizontal closure				
Ikiz et al. (2000) [9]	81	9.1%	Three-layer closure with polyglactin 910	4/6
Süslü et al. (2016) [19]	580	11.9%	Continuous suture, three-layer closure with polyglactin 910	5/6
Nitassi et al. (2016) [20]	14	21.4%	Not available	4/6
Govindasamy et al. (2018) [25]	3	0	Not available	4/6
Sansa-Perna et al. (2020) [28]	41	19.5%	Three-layers closure with polyglactin 910	5/6
Pooled estimates for fistular incidences				
T-shaped closure	795	RE = 19.9% (95% CI = 15.0-24.8), FE = 13.35% (95% CI =13.33-13.37)		
Vertical closure	232	RE = 16.1% (95% CI = 8.1-24.0), FE = 8.44% (95% CI =8.41-8.48)		
Horizontal closure	734	RE = 16.4% (95% CI = 13.3-19.5), FE = 12.03% (95% CI =12.00-12.05)		

**Table 2:** Summary of studies included for analysis of pooled estimates for fistular rates in salvage total laryngectomy. RF: random-effects model; FE: fixed-effects model; CI: confidence interval

Types of closure	Case	Fistula (%)	Surgical technique (suture of mucosa layer, number of layers, and suture material)	Quality assessment
T-shaped closure				
Virtaniemi et al. (2001) [10]	38	28.9%	Connell suture, two layers closure with Dexon	4/6
Markou et al. (2004) [11]	69	11.6%	Interrupted suture, two-layer closure	4/6
Busoni et al. (2015) [16]	189	29.1%	Single-layer closure with polyglactin 910	4/6
Süslü et al. (2015) [17]	5	60.0%	Continuous suture, three-layer closure with polyglactin 910	5/6
Walton et al. (2018) [24]	5	80.0%	Not available	4/6
Govindasamy et al. (2018) [25]	12	58.3%	Not available	4/6
Sansa-Perna et al. (2020) [28]	39	12.8%	Three-layer closure with polyglactin 910	5/6
Kitano et al. (2021) [29]	12	0	Continuous suture, two-layer closure with Polydioxanone	4/6
Vertical closure				
Mohamed et al. (2014) [14]	8	37.5	Connell sutures, two-layer closure with polyglactin 910	4/6
Walton et al. (2018) [24]	24	37.5%	Not available	4/6
Ogunkeyede et al. (2020) [27]	3	33.3%	Continuous suture, two-layer closure with polyglactin 910	4/6
Horizontal closure				
Ikiz et al. (2000) [9]	3	33.3	Three-layer closure with polyglactin 910	4/6
Süslü et al. (2015) [17]	146	11.6	Continuous suture, three-layer closure with polyglactin 910	5/6
Dulguerov et al. (2017) [23]	10	10%	Not available	3/6
Govindasamy et al. (2018) [25]	6	16.6%	Not available	4/6
Pooled estimates for fistular incidences				
T-shaped closure	357	RE = 35.1% (95% CI = 23.9-46.2), FE = 20.06% (95% CI =20.02-20.1)		
Vertical closure	35	RE = 36.1% (95% CI = 34.1-38.1), FE = 37.12% (95% CI =36.96-37.28)		
Horizontal closure	165	RE = 17.9% (95% CI = 13.0-22.8), FE = 11.8% (95% CI =11.76-11.85)		

From the included studies, 33-89% of the fistula occurred after primary total laryngectomy was resolved with conservative treatments. In salvage total laryngectomy, the success rates of fistula closure with conservative treatments were 0-29%.

**Figure 2:**
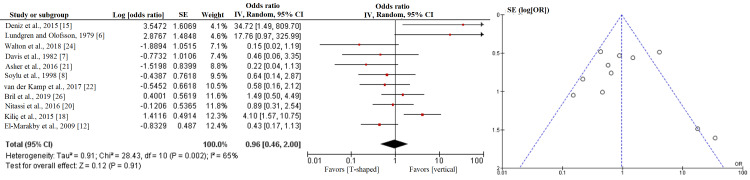
Forest and funnel plots of the analyses between T-shaped and vertical closures.

**Figure 3:**
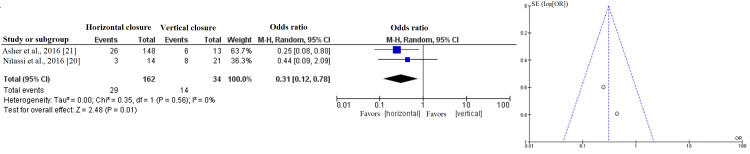
Forest and funnel plots of the analyses between horizontal and vertical closures.

**Figure 4:**
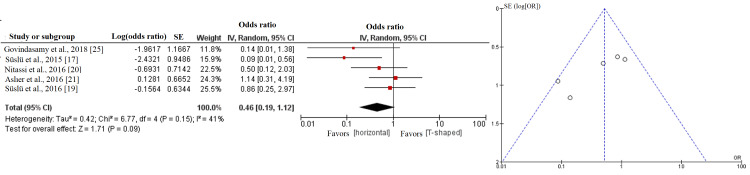
Forest and funnel plots of the analyses between horizontal and T-shaped closures.

Table 3 summarizes the analysis of the odds ratios for risk of fistula formation between the three closure shapes. Figures 2-4 show the forest and funnel plots for the analysis. The risk of fistula formation in T-shaped closure was comparable to that of vertical closure. The high level of heterogeneity reflected varied outcomes in the comparison between T-shaped and vertical closures.

**Table 3:** Analysis of pooled estimates for odds ratios of the occurrence of fistula. *Significant values. RF: random-effects model; FE: fixed-effects model; HET: heterogeneity

Study	Odds ratios (95% confidence interval)	Favors	Primary or salvage surgery	Quality assessment
Vertical vs T-shaped closures				
Vertical closure	1.00 (reference)	-	-	-
T-shaped closure	-	-	-	-
Pooled odds ratio (RE)	0.96 (0.46, 2.00), HET 65%	Vertical closure	-	-
Pooled odds ratio (FE)	0.97 (0.64, 1.45), HET 65%	Vertical closure	-	-
Lundgren and Olofsson (1979) [6]	17.76 (0.97, 325.99)	Vertical closure	Combined	6/9
Davis et al. (1982) [7]	0.46 (0.06, 3.35)	T-shaped closure	Primary	7/9
Soylu et al. (1998) [8]	0.64 (0.14, 2.87)	T-shaped closure	Combined	6/9
El-Marakby et al. (2009) [12]	0.43 (0.17, 1.13)	T-shaped closure	Combined	6/9
Deniz et al. (2015) [15]	34.72 (1.49, 809.70)*	Vertical closure	Primary	7/9
Kiliç et al. (2015) [18]	4.10 (1.57, 10.75)*	Vertical closure	Combined	6/9
Nitassi et al. (2016) [20]	0.89 (0.31, 2.54)	T-shaped closure	Primary	7/9
Aslıer et al. (2016) [21]	0.22 (0.04, 1.13)	T-shaped closure	Combined	6/9
van der Kamp et al. (2017) [22]	0.58 (0.16, 2.12)	T-shaped closure	Combined	6/9
Walton et al. (2018) [24]	0.15 (0.02, 1.19)	T-shaped closure	Combined	6/9
Bril et al. (2019) [26]	1.49 (0.50, 4.49)	Vertical closure	Combined	6/9
Vertical vs horizontal closures				
Vertical closure	1.00 (reference)	-	-	-
Horizontal closure	-	-	-	-
Pooled odds ratio (RE)	0.31 (0.12, 0.78)*, HET 0%	Horizontal closure	-	-
Pooled odds ratio (FE)	0.32 (0.12, 0.82)*, HET 0%	Horizontal closure	-	-
Nitassi et al. (2016) [20]	0.44 (0.09, 2.09)	Horizontal closure	Primary	7/9
Aslıer et al. (2016) [21]	0.25 (0.08, 0.80)*	Horizontal closure	Combined	6/9
T-shaped vs horizontal closures				
T-shaped closure	1.00 (reference)	-	-	-
horizontal closures	-	-	-	-
Pooled odds ratio (RE)	0.46 (0.19, 1.12), HET 41%	Horizontal closure	-	-
Pooled odds ratio (FE)	0.53 (0.29, 0.98)*, HET 41%	Horizontal closure	-	-
Süslü et al. (2015) [17]	0.09 (0.01, 0.56)	Horizontal closure	Salvage	7/9
Süslü et al. (2016) [19]	0.86 (0.25, 2.97)	Horizontal closure	Primary	7/9
Nitassi et al. (2016) [20]	0.50 (0.12, 2.03)	Horizontal closure	Primary	7/9
Aslıer et al. (2016) [21]	1.14 (0.31, 4.19)	T-shaped closure	Combined	6/9
Govindasamy et al. (2018) [25]	0.14 (0.01, 1.38)	Horizontal closure	Combined	6/9

Horizontal closure, when compared to vertical closure, was significantly associated with lower risk of fistula formation but had a nonsignificantly lower risk of fistula formation when compared to T-shaped closure. The low level of heterogeneity indicated that the majority of studies showed favorable outcomes toward horizontal closure.

Discussion

Pharyngocutaneous fistula is the most common complication encountered in the postoperative period following total laryngectomy. Surgical techniques during neopharyngeal reconstruction are crucial in preventing post-operative fistulas.

Several issues regarding surgical techniques for pharyngeal reconstruction have been studied. For example, recent studies have shown that the use of continuous sutures is associated with lower fistula rates than interrupted sutures in mucosal repair, and nonclosure of the pharyngeal constrictor muscle is not associated with a higher rate of fistula formation [2,30]. However, the relationship between shapes of pharyngeal closure and fistula outcome is not well-established and the study aims to address this.

This study shows that vertical closure yields similar fistula incidence rates and outcomes when compared with T-shaped closure. Both closure shapes have their advantages and disadvantages. The trifurcation in the T-shaped closure might increase the risk of fistula formation. However, the T-shaped closure causes less tension than the vertical closure in some defect shapes, which decreases the risk of fistula formation [22]. The surgeon's preference and experience between T-shaped and vertical closures might cause varying surgical outcomes which result in high heterogeneity of the analysis.

Swallowing is another aspect to be considered. Previous studies have reported that the swallowing function of horizontal and T-shaped closures was superior to that of vertical closure [22,31]. The etiology of the relationship between swallow function and pharyngeal repair techniques is not well understood. Dysphagia may be related to the diameter, outflow resistance, and function of the neopharynx [32]. Formation of a pseudo-diverticulum at the neopharynx is more frequently seen in laryngectomy patients closed with vertical closure and may cause dysphagia [32]. This diverticulum may result from surplus tissue or tension on the wound edges when applying vertical shape closure.

Horizontal closure seems to be the ideal closure shape for primary closure of the neopharyngeal reconstruction. Horizontal closure produces a relaxed and wide neopharynx, similar to T-shaped closure, but avoids trifurcation, similar to vertical closure. The results of this study also support the superior outcome of horizontal closure in fistula prevention. However, horizontal closure may have limited use because it is not suitable for vertically extended pharyngeal defects.

The following limitations should be considered in the analysis. First, none of the included studies were controlled trials. Other known risk factors for fistula formation, such as age, smoking, T-stage, tumor site, and nutritional status, might confound analysis outcomes.

Salvage surgery after concurrent chemoradiotherapy had higher complication and fistula rates than surgery after radiotherapy alone [33]. In the majority of studies, these factors were not strictly separated. This might affect reliability of analysis of fistula outcomes after salvage total laryngectomy.

Horizontal and vertical closures were usually performed in smaller pharyngeal defects. Therefore, bias related to the T-stage and tumor site might have occurred. Omitting nonEnglish-language studies might result in biased samples. Finally, although horizontal closure showed a significantly lower risk of fistula formation than vertical closure, only two studies were available for analysis. Thus, the results might be limited.

## Conclusions

The fistula outcomes between T-shaped and vertical closures were comparable. Horizontal closure had notably lower fistula incidence in salvage total laryngectomy and was associated with a lower risk of fistula formation when compared with T-shaped and vertical closures. These findings suggest that horizontal closure is the best closure shape for primary repair after total laryngectomy. However, bias in analysis may have occurred because of the lack of well-controlled studies.
